# Whole body MRI with Diffusion Weighted Imaging versus 18F-fluorodeoxyglucose-PET/CT in the staging of lymphomas

**DOI:** 10.1007/s11547-023-01622-9

**Published:** 2023-05-05

**Authors:** Francesca Maccioni, Giulia Alfieri, Giovanni Manfredi Assanto, Monica Mattone, Guido Gentiloni Silveri, Federica Viola, Alessandro De Maio, Viviana Frantellizzi, Alice Di Rocco, Giuseppe De Vincentis, Alessandro Pulsoni, Maurizio Martelli, Carlo Catalano

**Affiliations:** 1 Department of Radiological, Oncological and Anatomopathological Sciences, Sapienza University of Rome, Policlinico Umberto I Hospital, Viale Regina Elena 324, 00161 Rome, Italy; 2 Department of Translational and Precision Medicine, Sapienza University of Rome, Policlinico Umberto I Hospital, Via Benevento 6, 00161 Rome, Italy

**Keywords:** Lymphoma, Whole-body-Magnetic Resonance Imaging, Diffusion Weighted Imaging, 18F-fluorodeoxyglucose-PET/CT, Chemotherapy

## Abstract

**Purpose:**

To assess the diagnostic performance of Whole Body (WB)-MRI in comparison with 18F-Fluorodeoxyglucose-PET/CT (18F-FDG-PET/CT) in lymphoma staging and to assess whether quantitative metabolic parameters from 18F-FDG-PET/CT and Apparent Diffusion Coefficient (ADC) values are related.

**Materials and methods:**

We prospectively enrolled patients with a histologically proven primary nodal lymphoma to  undergo 18F-FDG-PET/CT and WB-MRI, both performed within 15 days one from the other, either before starting treatment (baseline) or during treatment (interim). Positive and negative predictive values of WB-MRI for the identification of nodal and extra-nodal disease were measured. The agreement between WB-MRI and 18F-FDG-PET/CT for the identification of lesions and staging was assessed through Cohen's coefficient k and observed agreement. Quantitative parameters of nodal lesions derived from 18F-FDG-PET/CT and WB-MRI (ADC) were measured and the Pearson or Spearman correlation coefficient was used to assess the correlation between them. The specified level of significance was *p* ≤ 0.05.

**Results:**

Among the 91 identified patients, 8 refused to participate and 22 met exclusion criteria, thus images from 61 patients (37 men, mean age 30.7 years) were evaluated. The agreement between 18F-FDG-PET/CT and WB-MRI for the identification of nodal and extra-nodal lesions was 0.95 (95% CI 0.92 to 0.98) and 1.00 (95% CI NA), respectively; for staging it was 1.00 (95% CI NA). A strong negative correlation was found between ADCmean and SUVmean of nodal lesions in patients evaluated at baseline (Spearman coefficient r_s_ = − 0.61, *p* = 0.001).

**Conclusion:**

WB-MRI has a good diagnostic performance for staging of patients with lymphoma in comparison with 18F-FDG-PET/CT and is a promising technique for the quantitative assessment of disease burden in these patients.

**Supplementary Information:**

The online version contains supplementary material available at 10.1007/s11547-023-01622-9.

## Background

Malignant lymphoma accounts for 5–6% of all malignancies and represents the third most common malignant tumor in children. To date, the prognosis of these patients has improved with a 5-year survival rate between 63 and 91% [1–3]. In patients with lymphoma, determination of the extent of the disease is critical to guide treatment decisions, and positron emission tomography with (18)F-Fluorodeoxyglucose (18F-FDG-PET/CT) is currently the diagnostic gold standard for disease staging [4]. 18F-FDG-PET/CT provides both structural and functional metabolic information to localize and characterize tumor burden [4–6]. Standardized uptake values (SUVs) of nodal and extra-nodal lesions derived from 18F-FDG-PET/CT measured both before initiating chemotherapy and during treatment correlate with tumor cellularity and aggressiveness [4, 7, 8]. However, 18F-FDG-PET/CT provides a significant dose of ionizing radiation. The risk of secondary malignancies due to radiation exposure may be of concern considering that a high percentage of lymphoma patients are young and that the efficacy of current chemotherapy regimens has led to increased and long life expectancy in these patients [9–12].

Whole body magnetic resonance imaging (WB-MRI) with Diffusion Weighted Imaging (DWI) is a promising, radiation-free technique that can be used for staging and follow-up of lymphoma and cancer diseases [13]. WB-MRI with DWI provides both anatomical and functional information; in fact, DWI-derived apparent diffusion coefficient (ADC) values quantify the degree of free movement of water molecules within tissues, which correlates with tumor cellularity. Several studies have reported significantly lower ADC values in lymphomas compared with normal lymph nodes and nodal sites of carcinoma [14]; moreover, ADC values increase after treatment in responder patients [15, 16]. To date, however, WB-MRI is still not routinely performed in patients with lymphoma because of the absence of standardized parameters for assessing disease burden and degree of response to treatment, compared with 18F-FDG-PET/CT [17].

Several studies have reported good to excellent agreement between 18F-FDG-PET/CT and WB-MRI for staging patients with lymphoma [13, 18–23]. Regarding the correlation between quantitative parameters derived from 18F-FDG-PET/CT and WB-MRI, some studies show a moderate to poor inverse correlation between SUVmax and ADCmean, while others report no correlation [24, 25]. On the other hand, only a few studies have investigated the association between the quantitative 18F-FDG-PET/CT parameters and WB-MRI ADC values. Therefore, the main purpose of this study was to investigate the performance of WB-MRI in disease staging compared with 18F-FDG-PET/CT and to evaluate the correlation between 18F-FDG-PET/CT parameters (SUVmax, SUVmean) with quantitative ADC values in patients with primary nodal HL and diffuse large B-cell lymphoma (DLBCL). A secondary aim was to investigate the changes in these parameters in the early post-treatment phase in a subgroup of patients with HL.

## Materials and methods

### Study design and participants

This is a single-center prospective study. Patients with a diagnosis of HL or DLBCL referred to the Hematology Unit of our University Hospital between May 2020 and July 2022 were consecutively evaluated. Patients who had to undergo a 18F-FDG-PET/CT examination for staging or follow-up purposes were considered for inclusion. Inclusion and exclusion criteria were assessed during the hematological visit. In the same visit, patients were informed regarding the possibility of being enrolled and a written informed consent was given by all participants before inclusion.

Inclusion criteria were: age 18 years or older; a histopathological proven diagnosis of HL or DLBCL in patients who were candidates to immuno-chemotherapy or who were already receiving immuno-chemotherapy (having undergone up to 2 treatment cycles). Exclusion criteria were: general contraindications for MRI, the presence of a concurrent malignancy other than lymphoma, women in the first trimester of pregnancy, interval between 18F-FDG-PET/CT and WB-MRI examinations greater than 15 days, one or more chemotherapy treatment sessions received during the time interval between 18F-FDG-PET/CT and WB-MRI examinations. Enrolled patients were scheduled for a WB- MRI examination within 15 days from the 18F-FDG-PET/CT examination. The Ethics Committee of Sapienza University of Rome approval was obtained for the study.

### WB-MRI and FDG-PET-CT examinations

WB-MRI examinations were performed at our institution, using a 3 T (Vida, Siemens Healthlineers) or a 1.5 T (Avanto, Siemens Healthlineers) MRI system. The protocol included coronal T1W Dixon (3 T) or VIBE (1.5 T) sequences, T2W half-Fourier multi-shot Turbo spin-echo, axial DWI with three b-values at 1.5 T (b = 50 s/mm^2^, b = 400 s/mm^2^ and b = 800 s/mm^2^ at 1.5 T) and with two b-values at 3 T ((b = 50 s/mm^2^, b = 800 s/mm^2^). A built-in body receiver coil was used. The details of the imaging protocol are shown in Supplemental Material - Table 1. All examinations were performed without using contrast media.

All the 18F-FDG-PET/CT examinations were performed at other institutions and patients were asked to provide images in digital form (DICOM format).

### WB-MRI image analysis

All the MRI images were analyzed on a Picture Archiving and Communication System workstation (INFINITT, INFINITT Healthcare). Two radiologists, one with 25-year experience and one with 5-year experience, blinded to clinical data and 18F-FDG PET/CT results, independently reviewed WB-MRI images. Discrepancies in measurements were resolved in consensus. A third doctor, a resident with 4-year experience, analyzed images to evaluate the inter-observer agreement. Disease staging was assessed using the Revised Staging System for Primary Nodal Lymphomas (Supplemental Material - Table 2) [4]. Nodal involvement was assessed in 10 nodal stations: cervical, supraclavicular, axillary, mediastinal, hilar, para-aortic, mesenteric, iliac, inguinal regions, and other regions (such as liver hilum) [26, 27]. Lymph nodes were considered involved when on axial images the largest diameter was greater than 1.5 cm or the short axis was greater than 1 cm or when they coalesced into large nodal masses, the DWI signal was higher than that of the spinal cord, or in the presence of central necrosis, regardless of the diameter [28].

For the evaluation of extra-nodal disease, one organ was considered involved when at least one of the following criteria was present: focal or diffuse alterations of signal intensity on T2W images, splenomegaly (greater than 13 cm in the maximum diameter); asymmetrical enlargement of bilateral organs (including salivary glands, lacrimal glands, tonsils); high signal intensity on DWI in extra-nodal organs (spleen, gallbladder, liver adrenal gland, prostate, testicles, penis, endometrium, adnexa, brain, spinal cord, salivary glands, tonsils, bone marrow).

**Fig. 1:**
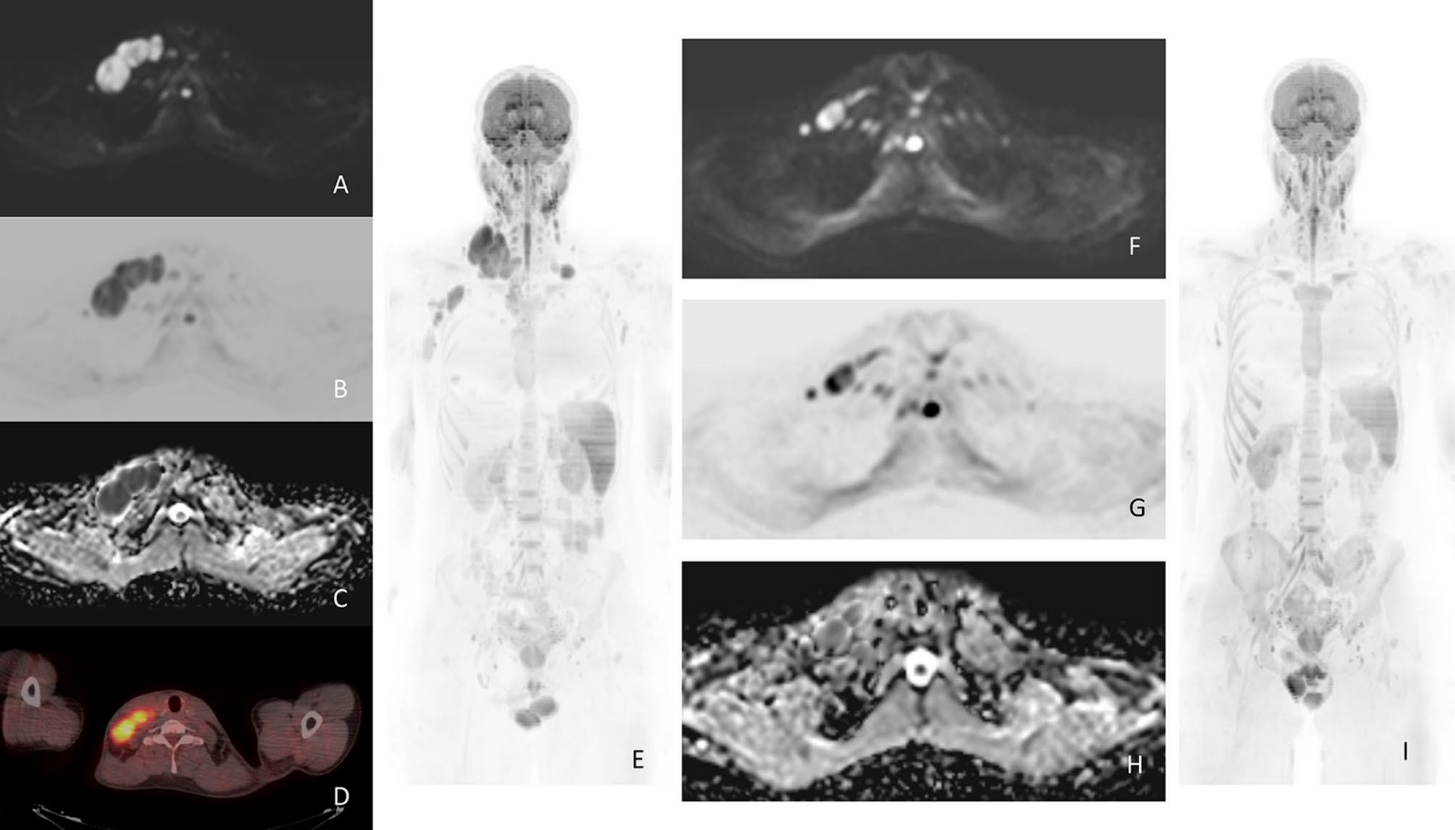
Baseline (A-E) and interim (F-I) Whole Body-MRI (WB-MRI) in a 31-year-old man with a mixed cellularity lymphoma and multiple confluent right supraclavicular nodal lesions at baseline that reduced in size at interim. At baseline, the lesions appear as hyperintense in the axial Diffusion Weighted image (b = 800 s/mm^2^) (**A)** and hypointense in the axial grayscale inverted Diffusion Weighted image (b = 800 s/mm^2^) (**B**). **C** On the baseline WB-MRI Apparent Diffusion Coefficient (ADC) map, the lesions appear as hypointense. **D** Baseline PET/CT shows large confluent nodal lesions in the same region with a high SUV value. **E** Coronal grayscale inverted MIP DW-MRI shows the same lesions that appear hypointense. At interim, the lesions appear significantly reduced in size and less hyperintense than baseline in the axial Diffusion Weighted image (b = 800 s/mm^2^) and (**F**) less hypointense than baseline in the axial grayscale inverted Diffusion Weighted image (b = 800 s/mm^2^) (**G**). **H** On the interim WB-MRI Apparent Diffusion Coefficient (ADC) map, the same lesions appear more hyperintense than baseline. **I** Coronal grayscale inverted MIP DW-MRI shows minimal residual disease

For the quantitative analysis, the largest lesion (a single lymph node or a single mass when coalesced) in each nodal region was selected. For the assessment of ADC values, the target lesions were identified on axial ADC maps. Lesions located within areas with visible motion artifacts were excluded. For the measurement of the ADC values of a single lesion (a single lymph node or a single mass when coalesced), ROIs were manually placed on each slice in which the lesion was visible, avoiding necrotic areas. The system automatically provided the minimum (ADCmin) and mean (ADCmean) values (Supplemental Material - Figure 1). The ADCmean value of the entire lesion was the mean of all the ADCmean values measured in each slice. The ADCmin value of the entire lesion was the minimum ADCmin value among each slice.

### 18F-FDG-PET/CT image analysis

All the 18F-FDG-PET/CT images at baseline and interim were analyzed using the software syngo.via (Siemens Healthlineers). A nuclear medicine doctor with 15-year experience and a resident with 4-year experience, blinded to clinical data, independently reviewed 18F-FDG-PET/CT images. Discrepancies in measurements were resolved in consensus. Disease staging was assessed using the Revised Staging System for Primary Nodal Lymphomas [4]. Nodal involvement was assessed at the level of the 10 nodal stations considered for the WB-MRI analysis. Disease presence was scored either positive (uptake above surrounding background in a location incompatible with normal physiological activity) or negative for 10 nodal and all extra-nodal stations. The largest lesion (a single lymph node or a single mass when coalesced) in each nodal region was selected for the quantitative analysis. Using a semiautomatic segmentation method, metabolic parameters were measured in the single volume of interest (VOIs). VOIs were placed in each nodal lesion or in each nodal mass when coalesced and the software automatically provided SUVmax and SUVmean (Supplemental Material - Figure 1).

### Statistical analysis

Descriptive statistics were performed to summarize the patient's characteristics: mean (± standard deviation) or median (interquartile range) were used, as appropriate. The Kolmorgov-Smirnov test was used to assess normality. Positive (PPV) and negative (NPV) predictive values of 18F-FDG-PET/CT and WB-MRI for the identification of nodal- and extra-nodal lesions was measured in comparison to 18F-FDG-PET/CT as the reference standard. The agreement between 18F-FDG-PET/CT and WB-MRI for the identification of nodal and extra-nodal involvement and staging was evaluated through kappa coefficient and observed agreement. The analysis of ADC and 18F-FDG-PET/CT-derived parameters was based on per-lesion datasets. Inter-observer reproducibility of baseline and interim- ADC values were determined as mean absolute difference (bias) and 95% CIs of the mean difference (limits of agreement), according to the Bland–Altman method. 18F-FDG-PET/CT-derived parameters and ADC values were compared between patients examined at baseline and interim through Mann-Withney test. The Pearson correlation or Spearman rank correlation tests were used to estimating the association between 18F-FDG-PET/CT parameters and ADC, as appropriate. Bonferroni-corrected post-hoc tests were used for the correlation analysis. In the subgroup of patients who underwent both baseline and interim examinations WB-MRI and 18F-FDG-PET/CT-derived parameters were compared between baseline and interim examination using the Wilcoxon matched-pairs signed rank test. The Spearman rank correlation was used to estimate the association between 18F-FDG-PET/CT-derived and WB-MRI-derived parameters, considering both interval change and rate of change following treatment. Bonferroni-corrected post-hoc tests were used for the correlation analysis. The specified level of significance was *p* ≤ 0.05 for all tests. All statistical tests were performed using Prism ver. 9.0 (GraphPad Software, Inc., San Diego, California).

## Results

### Patients population

Between May 2020 and July 2022, a total of 91 patients were found eligible for inclusion. Thirty patients were excluded: in thirteen patients the time interval between WB-MRI and 18F-FDG-PET/CT exceeded 15 days, 9 patients underwent a chemotherapy treatment session between the WB-MRI and 18F-FDG-PET/CT examinations and 8 patients refused to participate. 

A total of 61 patients were evaluated (37 men, 24 women, mean age 30.7 ± 18.25 years). Thirty-four patients were treatment naïve (baseline) and 27 patients were under treatment (interim). Among the 34 treatment naïve patients, 28 had HL and 6 had DLBCL, whereas all the 27 patients under treatment had HL (25 patients were receiving doxorubicin hydrochloride, bleomycin sulfate, vinblastine sulfate, and dacarbazine (ABVD), whereas 2 received brentuximab vedotin, doxorubicin, vinblastine, and dacarbazine (A + AVD)). Baseline characteristics are shown in Table 1.

**Table 1:** Patients’ baseline characteristics

Total number – n	61
Men – n (%); Women – n (%)	37 (61%); 24 (39%)
Mean age (± SD)—y	30.70 ± 18.25
Time-point of examinations	
Baseline – n (%)	34 (56)
Interim – n (%)	27 (44)
**Histology – n (%)**	
Nodular Sclerosing	12 (20)
Mixed cellularity HL	7 (11)
Classic HL	36 (59)
DLBCL	6 (10)
**WB-MRI parameters**	
**ADCmin (mm** ^**2**^**/s)**	
All patients—mean ± SD	0.764 ± 0.321 × 10^–3^
Baseline—median (IQR)	0.506 (0.409–0.569) × 10^–3^
Interim—median (IQR)	0.991 (0.912–1.049) × 10^–3^
**ADCmean (mm**^**2**^**/s)**	
All patients—mean ± SD	0.974 ± 0.384 × 10^–3^
Baseline—median (IQR)	0.646 (0.599–0.707) × 10^–3^
Interim—median (IQR)	1.261 (1.080–1.402) × 10^–3^
**18F-FDG-PET/CT parameters**	
**SUVmax – median (IQR)**	
All patients	5.2 (2.5–11.7)
Baseline	15.0 (13.1–17.8)
Interim	2.3 (1.0–3.2)
**SUVmean – median (IQR)**	
All patients	3.1 (2.6–4.0)
Baseline	5.1 (4.8–5.9)
Interim	2.0 (1–2.4)

HL = Hodgkin Lymphoma, DLBCL = Diffuse Large B Cell Lymphoma, WB-MRI = Whole body Magnetic Resonance Imaging, ADCmin = minimum Apparent Diffusion Coefficient, ADCmean = mean Apparent Diffusion Coefficient, 18F-FDG-PET/CT = 18Fluoro-Fluorodesoxyglucose-PET/CT, SUVmax = maximum Standardized Uptake Value, SUVmean = mean Standardized Uptake Value

The median interval between baseline 18F-FDG-PET/CT and WB-MRI examinations was 7 days (range 1–12 days).

### Staging

A total of 610 nodal regions were analyzed; among these, 132/610 (22%) were considered positive at 18F-FDG-PET/CT and 478/610 (78%) as negative. At WB-MRI, 127/610 (21%) were positive and 483/610 (79%) negative; 3/127 regions (2%) considered as positive at WB-MRI were negative at 18F-FDG-PET/CT, considered as false positive (1 cervical, 1 mesenteric and 1 lesion classified as “other regions” that was located at the hepatic hilum), whereas 124/ 132 were true positive (94%). Furthermore, WB-MRI did not detect 8/132 PET positive regions (6%, 1 axillary, 1 mediastinal, and 6 hilar regions), thus considered WB-MRI false negatives.

For the assessment of nodal involvement in all regions, the PPV and NPV of WB-MRI were respectively 0.98 (95% CI 0.93 to 0.99) and 0.98 (95% CI 0.97 to 0.99). The observed agreement between 18F-FDG-PET/CT and WB-MRI for all nodal regions was 0.98 (599/610, 95% CI 0.97 to 0.99); Cohen’s kappa coefficient was 0.95 (95% CI 0.92 to 0.98). Additional results are shown in Table 2(a).

**Table 2:** Positive predictive value (PPV), negative predictive value (NPV) of WB-MRI, Cohen’s kappa and observed agreement between WB-MRI and 18F-FDG-PET/CT for the identification of (a) nodal lesions, (b) extra-nodal lesions

	PPV (95% CI)	NPV (95% CI)	Kappa (95% CI)	Observed agreement n/n (95% CI)
*(a) Nodal lesions*				
Cervical	0.93 (0.70 to 1.00)	1.00 (0.92 to 1.00)	0.96 (0.87 to 1.00)	0.98 (60/61, 0.95 to 1)
Supraclavicular	1.00 (0.85 to 1.00)	1.00 (0.91 to 1.00)	1.00 (NA)	1.00 (61/61, NA)
Axillary	1.00 (0.72 to 1.00)	0.98 (0.90 to 1.00)	0.94 (0.83 to 1.00)	0.98 (60/61, 0.95 to 1)
Mediastinal	1.00 (0.89 to 1.00)	0.97 (0.84 to 1.00)	0.97 (0.90 to 1.00)	0.98 (60/61, 0.95 to 1)
Hilar	1.00(0.87 to 1.00)	0.833 (0.68 to 0.92)	0.80 (0.66 to 0.95)	0.90 (55/61, 0.83 to 0.97)
Para-aortic	1.00 (0.72 to 1.00)	1.00 (0.93 to 1.00)	1.00 (NA)	1.00 (61/61, NA)
Mesenteric	0.86 (0.49 to 0.99)	1.00 (0.93 to 1.00)	0.91 (0.75 to 1.00)	0.98 (60/61, 0.95 to 1)
Iliac	1.00 (0.51 to 1.00)	1.00 (0.94 to 1.00)	1.00 (NA)	1.00 (61/61, NA)
Inguinal	1.00 (0.17 to 1.00)	1.00 (0.94 to 1.00	1.00 (NA)	1.00 (61/61, NA)
Other	0.50 (0.03 to 0. 97)	1.00 (0.94 to 1.00)	0.66 (0.04 to 1.00)	0.98 (60/61, 0.95 to 1)
All nodal regions combined	0.98 (0.93 to 0.99)	0.98 (0.97 to 0.99)	0.95 (0.92 to 0.98)	0.98 (599/610, 0.97 to 0.99)
*(b) Extra-nodal lesions*				
Liver	1.00 (0.44 to 1.00)	1.00 0.94 to 1.00)	1.00 (NA)	1.00 (61/61, NA)
Spleen	1.00 (0.65 to 1.00)	1.00 (0.93 to 1.00)	1.00 (NA)	1.00 (61/61, NA)
Bone marrow	1.00 (0.65 to 1.00)	0.98 (0,93 to 1.00)	1.000 (NA)	1.00 (61/61, NA)
All extra-nodal regions combined	1.00 (0.82 to 1.00)	1.00 (0.82 to 1.00)	1.00 (NA)	1.00 (183/183, NA)

Both 18F-FDG-PET/CT and WB-MRI identified extra-nodal involvement in the liver, spleen, and bone marrow with the same accuracy. Of 61 liver regions, 61 splenic regions, and 61 bone marrow regions analyzed, 17/183 (9%) were considered positive and 166/183 (90%) as negative at both 18F-FDG-PET/CT and at WB-MRI. For the assessment of extra-nodal involvement in all regions, the PPV and NPV of WB-MRI were 1.00 (95% CI 0.82 to 1.00) and 1.00 (95% CI 0.82 to 1.00), respectively. The observed agreement between 18F-FDG-PET/CT and WB-MRI for all extra-nodal regions was 1.00 (183/183, 95% CI NA); Cohen's kappa coefficient was 1.00 (95% CI NA). Additional results are shown in Table 2(b).

With regard to the staging, according to the reference standard evaluation, 13/61 (21%) patients were stage 1, 27/61 (44%) stage 2, 11/61 (18%) stage 3, and 10/61 (16%) stage 4. The agreement between 18F-FDG-PET/CT and WB-MRI for staging was 1.00 (95% CI NA).

### Quantitative analysis

Based on the Bland–Altman plot of measurements of two observers, ADCmin value measurement exhibits a bias of -0,011 and 95% limits of agreement of − 0.16 × 10^−3^ mm^2^/s and 0.14 × 10^−3^ mm^2^/s and ADCmean value a bias of − 0.0050 and 95% limits of agreement of − 0.18 × 10^−3^ mm^2^/s and 0.16 × 10^−3^ mm^2^/s.

The mean ADCmin value of all nodal lesions analyzed was 0.764 ± 0.321 × 10^−3^ mm^2^/s, and the mean ADCmean value of all nodal lesions analyzed was 0.974 ± 0.384 × 10^−3^ mm^2^/s. ADC and SUV values in the subgroups are reported in Table 1.

The median SUVmax value was 5.2 (IQR 2.5–11.7) and the median SUVmean 3.1 (IQR 2.6–4.0). A significant difference was found between baseline and interim ADCmin (*p* < 0.001) and ADCmean (*p* < 0.001) values, with higher values at interim. A significant difference was found between baseline and interim SUVmax (*p* < 0.001) and SUVmean (*p* < 0.001) values, with higher values at baseline. A negative correlation between ADCmean and SUVmean (Spearman correlation coefficient r_s= _− 0.61, *p* = 0.001) was found in naïve patients, whereas no correlation was found between ADCmean and SUVmean values at interim. 18F-FDG-PET/CT parameters were not correlated with ADCmin (p > 0.05).

### Subgroup analysis: variation of 18F-FDG-PET/CT and WB-MRI parameters during treatment in patients with Hodgkin lymphoma

Eight participants with HL underwent both baseline and interim examinations, following 2 treatment cycles with ABVD (Fig. 1). At the interim examination, all 8 patients had a complete response, based on the Lugano classification [4]; the Deauville score was 1 in 4/8 (50%) patients, 2 in 2/8 (25%) patients and 3 in 2/8 (25%) patients. The median interval between baseline 18F-FDG-PET/CT and WB-MRI examinations was 5 days (range 1–8 days) whereas between interim examinations was 8 days (range 3–12 days).

**Fig. 2:**
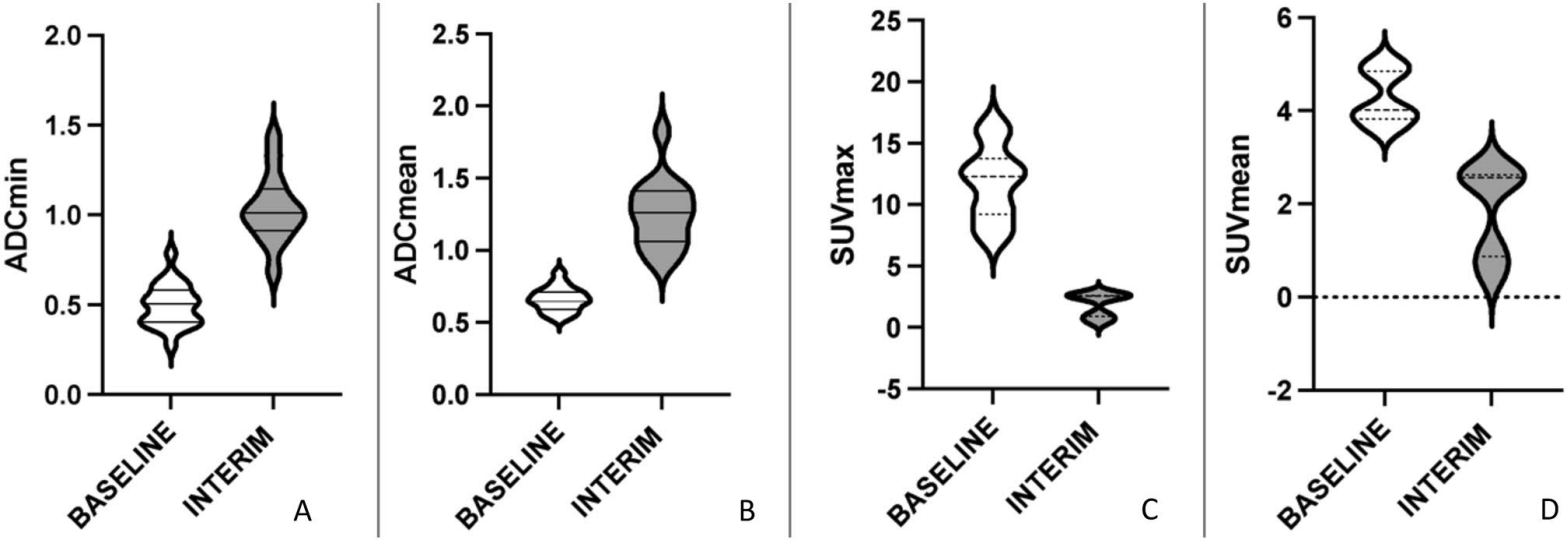
WB-MRI and 18F-FDG-PET/CT parameter change at interim from baseline in patients with Hodgkin lymphoma. Median ADCmin value was 0.440 (IQR 0.399–0.518) mm^2^/s at baseline and 0.970 (IQR 0.889–1.101) × 10^−3^ mm^2^/s at interim (median of differences = 0.524 × 10^−3^ mm^2^/s, *p* = 0.0005 (**A**). Median ADCmean value was 0.634 (IQR 0.589–0.668) × 10^−3^ mm^2^/s at baseline and 1.221 (IQR 1.017–1.266) × 10^−3^ mm^2^/s at interim (median of differences = 0.645 × 10^−3^ mm^2^/s, *p* = 0.002 (**B**). Regarding 18F-FDG-PET/CT values, median SUVmax values changed from 12.29 (IQR 9.73–13.00) at baseline to 2.64 (IQR 1.02–2.65) at interim (median of differences = − 10.43, *p* = 0.002, (**C**), SUVmean from 4.02 (IQR 3.9–4.8) at baseline to 2.1 (IQR 1–2.62) at interim (median of differences =− 2.17, *p* = 0.002, (**D**)

A total of 25 nodal lesions were analyzed. In this subgroup of patients, median ADCmin value at baseline was 0.440 (IQR 0.399–0.518) mm^2^/s, whereas at interim it was 0.970 (IQR 0.899–1.101) × 10^−3^ mm^2^/s (median of differences = 0.524 × 10^−3^ mm^2^/s, p = 0.0005) (Fig. 2a). Median ADCmean value at baseline was 0.634 (IQR 0.586–0.668) × 10^−3^ mm^2^/s, whereas at interim it was 1.221 (IQR 1.017–1.266) × 10^−3^ mm^2^/s (median of differences = 0.645 × 10^−3^ mm^2^/s, *p* = 0.002) (Fig. 2b). Regarding 18F-FDG-PET/CT values, median SUVmax values changed from 12.3 (IQR 9.73–13) to 2.7 (IQR 1.0–2.7) (median of differences = − 10.4, *p* = 0.002) (Fig. 2c), SUVmean values changed from 4.0 (IQR 3.9–4.8) to 2.1 (IQR 1.0–2.6, median of differences = − 2.2, *p* = 0.002) (Fig. 2d).

A strong negative correlation between ADCmean with SUVmax (Spearman correlation coefficient r_s_ = − 0.83, *p* < 0.0001) interval changes from baseline was found. No significant correlation was found between ADCmin and SUV (SUVmean and SUVmax) interval change. No correlation was found between SUV and ADC change rates (*p* > 0.05).

## Discussion

Our study demonstrates an excellent agreement between 18F-Fluorodesoxyglucose-PET/CT (18F-FDG-PET/CT) and Whole body-MRI (WB-MRI) with Diffusion Weighted Imaging (DWI) in the staging of patients with lymphoma, particularly for the assessment of nodal (Cohen’s kappa coefficient 0.95, 95% CI 0.92 to 0.98) and extra-nodal (Cohen’s kappa coefficient 1.00, 95% CI NA) disease; the highest amount of discrepancies between the two modalities was found in the pulmonary hilar region.

Regarding the quantitative analysis, a significant correlation was observed between baseline ADCmean and SUVmean values of lymph node lesions (*r* = − 0.61, * p* = 0.001). In the subgroup of eight patients with HL following the first treatment cycles (interim), the variation of ADCmean values was instead correlated with the variation of SUVmax values (*r* = − 0.83, *p* < 0.0001), whereas no correlation was found with SUVmean.

Our results are in line with previous literature data that have shown a good to excellent concordance of WB-MRI and 18F-FDG-PET/CT for the evaluation of nodal and extra-nodal disease [13, 18–22]. The discrepancies found between the two modalities in the pulmonary hilar regions can be explained by the proximity of this region to the lungs and heart, where artifacts secondary to cardiac and respiratory motion can affect the MRI assessment of nodal involvement. Notably, in all cases, the discrepancies in nodal and extra-nodal region identification did not affect the final evaluation of disease stage.

In our experience, the relatively low sensitivity of DWI in the assessment of spleen lesions [29], was overcome by combining it with morphological data (volume and signal inhomogeneity on T1 and T2 weighted sequences), thus achieving a high final accuracy of 100% (observed agreement 1.00).

Previous studies performed in patients with lymphoma reported controversial results regarding the correlation between SUV and ADC values measured on WB-MRI [24, 25]. Although the SUVmax is the most used and easy-to-use parameter in 18F-FDG-PET/TC, it does not reflect the metabolic activity of an entire lesion but measures a single-voxel value representing the most intense FDG uptake of a tumor [7]. In contrast, ADCmean values reflect cellularity inside an entire ROI. For these reasons, likely, at baseline, the ADCmean was strongly correlated to the SUVmean, which is the average SUV within a VOI [28]. No correlation was found, instead, when considering the SUVmean and ADCmean values of patients evaluated at interim and when considering together baseline and interim values (*p* > 0.05); one explanation could be that chemotherapy affects these quantitative parameters in different ways.

Our results in the subgroup of 8 patients with HL are in line with literature data, that have demonstrated an increase of ADC values in patients effectively responding to chemotherapy, as well as a reducion in SUVmax values [13]; in fact, in those patients, we found a strong correlation between the interval change of ADCmean with the interval change of SUVmax.

We can conclude that, although 18F-FDG-PET/CT and WB-MRI parameters reflect different biological processes, and many different factors contribute to tumor metabolism (as assessed by 18F-FDG-PET/CT) and tumor tissue composition (as assessed by ADC), the chemotherapy-induced changes in SUVmax and ADC values are related.

This study has several limitations. First of all, the inhomogeneity of the patients' population, which included naïve patients and patients under treatment. As a second limitation, 18F-FDG-PET/TC were performed at different institutions; third, WB-MRI was performed using two different systems (1.5 T and 3 T). Fourth, the gold standard was based on 18F-FDG-PET/CT, which, however, remains the best option to evaluate the diagnostic performance of WB-MRI due to the lack of an absolute gold standard (biopsy) for all lesions.

In conclusion, in this prospective study of 61 patients with Hodgkin and non-Hodgkin lymphoma, we found a high diagnostic performance of Whole-Body-MRI with DWI for both nodal and extra-nodal lesion identification and staging, in comparison with 18F-Fluorodeoxyglucose-PET/CT. Moreover, a strong correlation was found between baseline metabolic activity, as measured by the mean Standardized Uptake Values of nodal lesions, and tissue cellularity, as measured by the Apparent Diffusion Coefficient values. WB-MRI represents a promising technique for staging and quantifying disease activity in patients with Hodgkin and non-Hodgkin lymphoma. Unfortunately, there is still limited availability of adequate MRI hardware (especially whole-body coils) and software for WB-MRI examinations, especially at smaller imaging centers. However, a similar low availability is also experienced for PET-CT in our country. Based on these preliminary results, it is desirable that in the future WB-MRI with DWI will be increasingly used in staging lymphomas, before and during treatment, in place of 18F-FDG-PET/CT, especially in younger patients, to limit their radiation exposure. New clinical guidelines are desirable and expected to definitively introduce this imaging technique in the clinical evaluation of lymphomas. Further studies will be necessary to establish whether quantitative WB-MRI with DWI can discriminate the depth of treatment response to chemotherapy and whether it can provide prognostic information in comparison with 18F-FDG-PET/CT.

## Supplementary Information

Below is the link to the electronic supplementary material.

Supplementary file1 (Docx 3802 kb)
