# Supplementary material for: Whole body MRI with Diffusion Weighted Imaging versus 18F-fluorodeoxyglucose-PET/CT in the staging of lymphomas
Source: Radiol Med. 2023 May 5;128(5):556–64. doi: 10.1007/s11547-023-01622-9 (PMC10182138; doi:10.1007/s11547-023-01622-9)

**Supplemental material:**

# Supplemental Table 1 – Whole body MRI scan parameters

|  | Dixon/Vibe-T1w | HASTE-T2w | DWI |
| --- | --- | --- | --- |
| Repetition time (ms) | 4 (3T), 5.7 (1.5 T) | 1400 (3T), 1000 (1.5T) | 11000 (3T), 13500 (1.5T) |
| Inversion Time | - | - | 240 (3T), 180 (1.5T) |
| Echo time (ms) | 1 (in), 2.46 (opp) (3T), 3 (1.5T) | 86 (3T), 83 (1.5T) | 52 (3T), 76 (1.5T) |
| Slice thickness (mm) | 3,5 | 5 | 5 |
| Slice gap (mm) | 1 | 1 | 0 |
| Field of View (mm^2^) | 420x106 (3T), 400 x 325 (1.5T) | 400x100 (3T), 350x245  (1.5T) | 430x80 (3T), 382x286 (1.5T) |
| Acquisition matrix | 320x238 (3T), 256x146 (1.5T) | 368x294 (3T), 256x146  (1.5T) | 110x88 (3T), 192x115 (1.5T) |
| Cranio-caudal coverage | 115,5 | 115,5 | 115,5 |
| Plane | Coronal | Axial | Axial |
| B-values (s/mm^2^) | - | - | 50, 800 (3T),  50, 400, 800 (1.5T) |
| Total scan time (min) | 3-4 | 7-8 | 16-30 |

HASTE = Half Fourier Single-shot Turbo-spin Echo

# Supplemental Table 2 – Revised Staging System for Primary Nodal Lymphomas

| **Stage** | **Involvement** | **Extranodal (E) Status** |
| --- | --- | --- |
| **Limited** |  |  |
| **I** | One node or a group of adjacent nodes | Single extranodal lesions without nodal involvement |
| **II** | Two or more nodal groups on the same side of the diaphragm | Stage I or II by nodal extent with limited contiguous extranodal involvement |
| **Bulky** | II as above with “bulky” disease | Not applicable |
| **Advanced** |  |  |
| **III** | Nodes on both sides of the diaphragm; nodes above the diaphragm with spleen involvement | Not applicable |
| **IV** | Additional noncontiguous extralymphatic involvement | Not applicable |

**Supplemental Figure 1 - Measurement of 18F-FDG-PET/CT and WB-MRI parameters.** Whole Body-MRI (WB-MRI) and 18F-Fluorodeoxyglucose-PET/CT (18F-FDG-PET/CT) in a 29-year-old woman with classic Hodgkin lymphoma and large confluent retroperitoneal nodal lesions (A) On axial Diffusion Weighted image (b=800 s/mm^2^) the lesions have hyperintense signal. (B) On the WB-MRI Apparent Diffusion Coefficient (ADC) map, a ROI was placed on the largest lesion in each slice in which it was visible, avoiding necrotic areas and the system automatically provided the minimum (ADCmin) and mean (ADCmean) values. (C) On 18F-FDG-PET/CT examination, using a semiautomatic segmentation method, metabolic parameters were measured in the volume of interest (VOI). VOI was placed on the largest lesion in each involved region and the software automatically provided SUVmax and SUVmean.


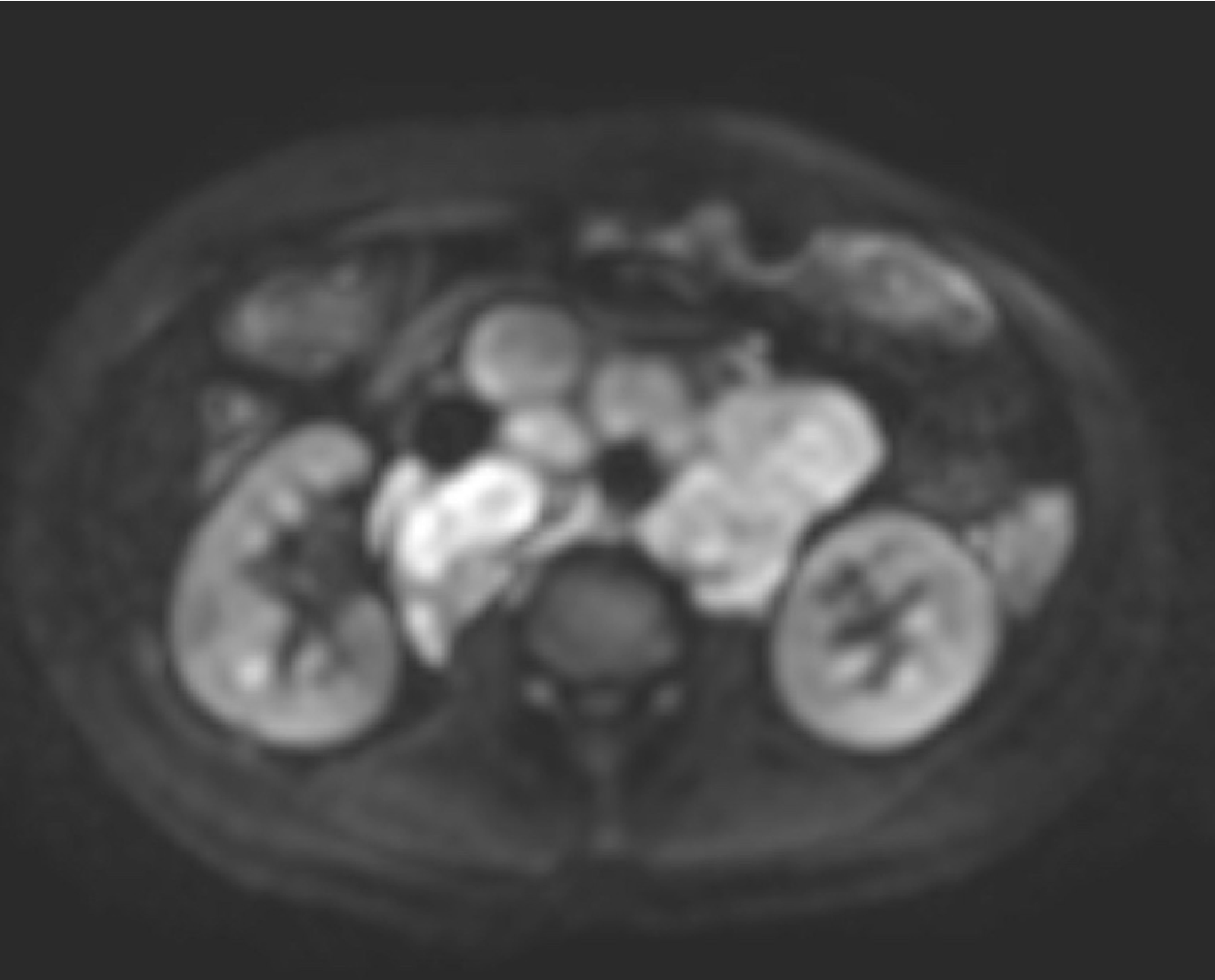

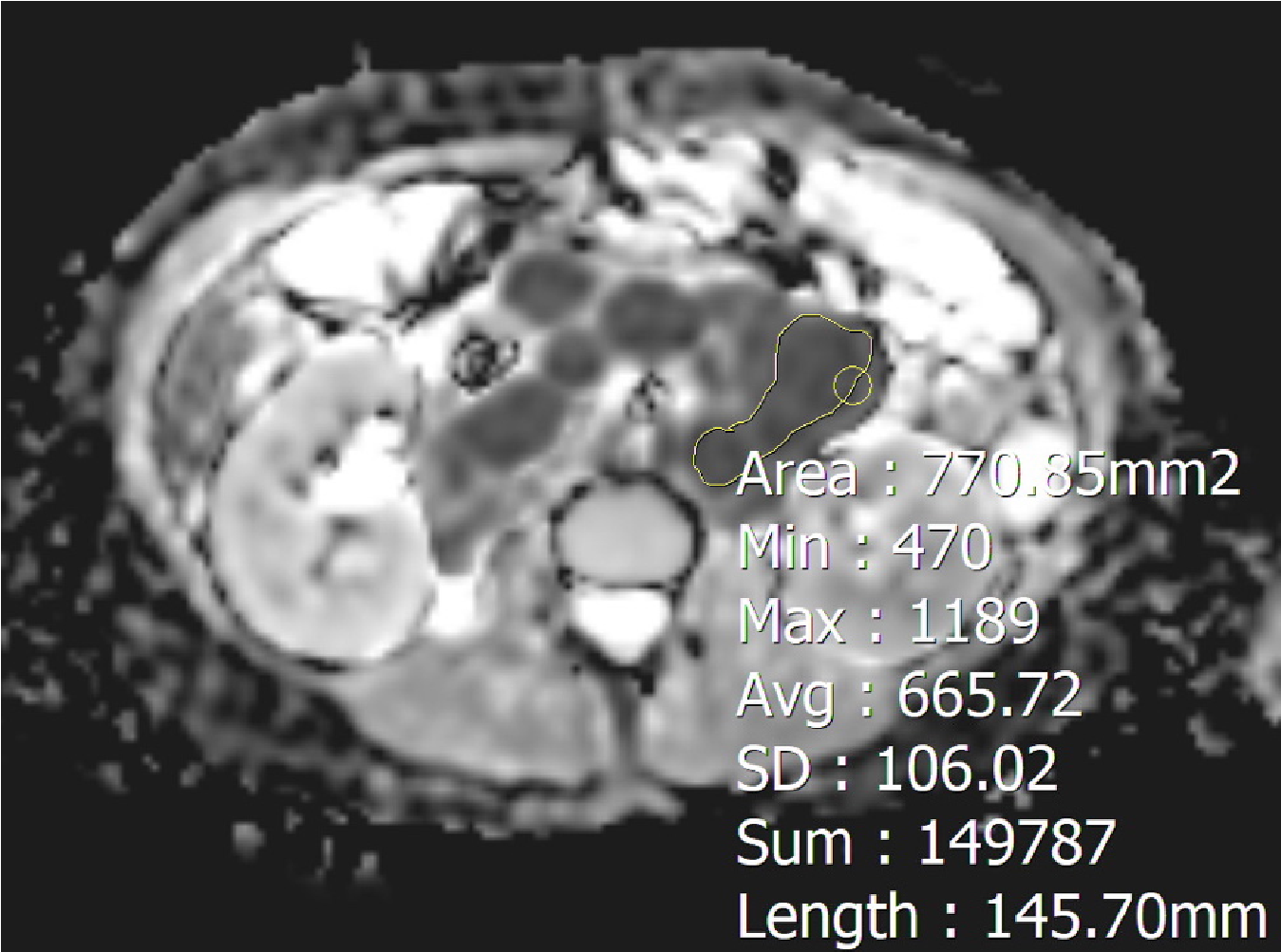

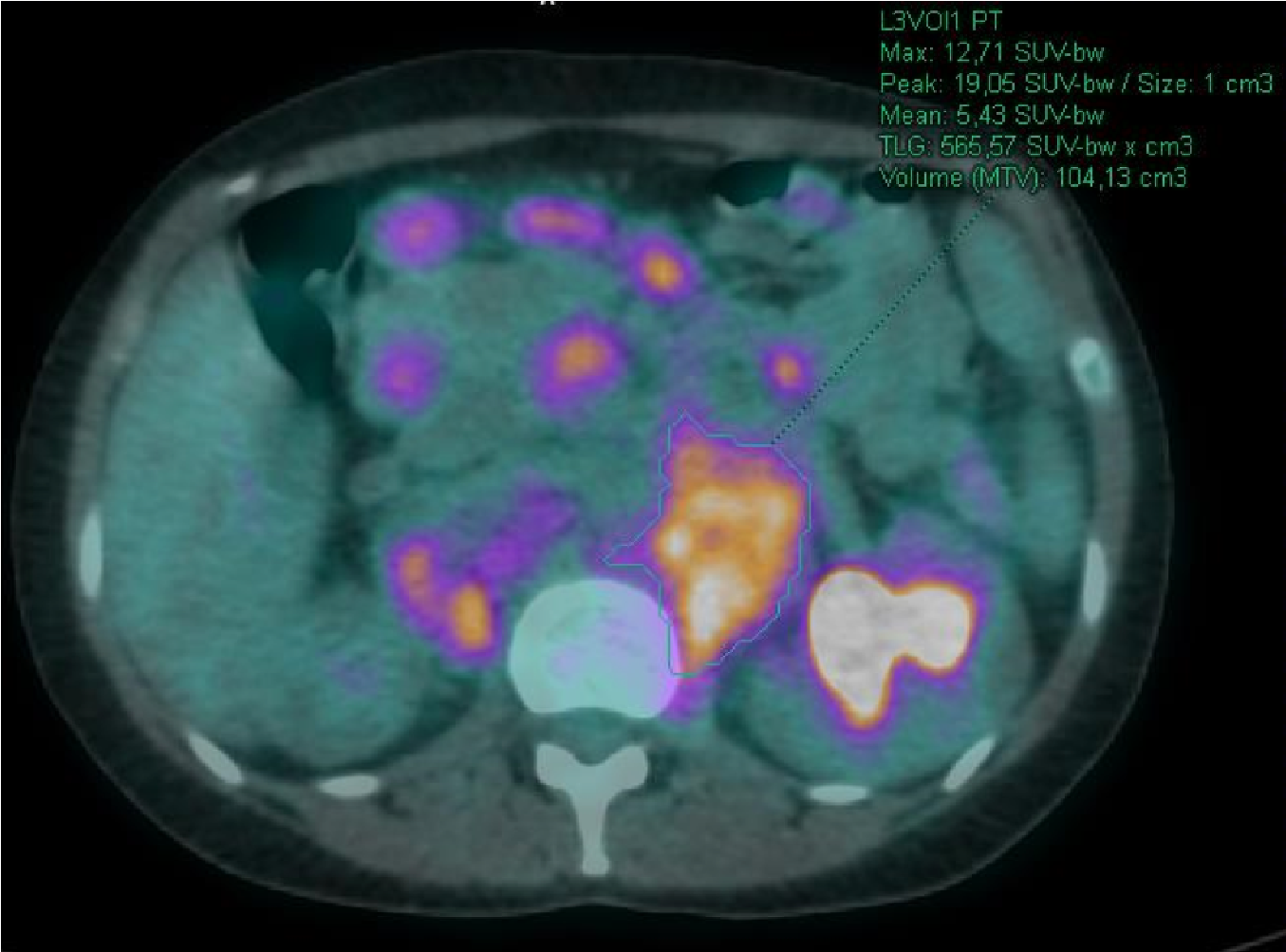

Supplement: Supplementary file 1 — Supplementary file1 (Docx 3802 kb) [file 11547_2023_1622_MOESM1_ESM.docx]
